# Nuclear factor E2-related factor 2 (NRF2) deficiency accelerates fast fibre type transition in soleus muscle during space flight

**DOI:** 10.1038/s42003-021-02334-4

**Published:** 2021-06-24

**Authors:** Takuto Hayashi, Takashi Kudo, Ryo Fujita, Shin-ichiro Fujita, Hirona Tsubouchi, Sayaka Fuseya, Riku Suzuki, Michito Hamada, Risa Okada, Masafumi Muratani, Dai Shiba, Takafumi Suzuki, Eiji Warabi, Masayuki Yamamoto, Satoru Takahashi

**Affiliations:** 1 Laboratory Animal Resource Center in Transborder Medical Research Center, and Department of Anatomy and Embryology, Faculty of Medicine, University of Tsukuba, Ibaraki, Japan; 2 Doctoral Program in Biomedical Sciences, Graduate School of Comprehensive Human Sciences, University of Tsukuba, Ibaraki, Japan; 3 Divsion of Regenerative Medicine, Transborder Medical Research Center, Faculty of Medicine, University of Tsukuba, Ibaraki, Japan; 4 Department of Genome Biology, Transborder Medical Research Center, Faculty of Medicine, University of Tsukuba, Ibaraki, Japan; 5 Ph.D. Program in Human Biology, School of Integrative and Global Majors, University of Tsukuba, Ibaraki, Japan; 6 JEM Utilization Center, Human Spaceflight Technology Directorate, JAXA, Ibaraki, Japan; 7 Department of Medical Biochemistry, Tohoku University Graduate School of Medicine, Sendai, Japan

**Keywords:** Transcriptomics, Skeletal muscle

## Abstract

Microgravity induces skeletal muscle atrophy, particularly in the soleus muscle, which is predominantly composed of slow-twitch myofibre (type I) and is sensitive to disuse. Muscle atrophy is commonly known to be associated with increased production of reactive oxygen species. However, the role of NRF2, a master regulator of antioxidative response, in skeletal muscle plasticity during microgravity-induced atrophy, is not known. To investigate the role of NRF2 in skeletal muscle within a microgravity environment, wild-type and *Nrf2-*knockout (KO) mice were housed in the International Space Station for 31 days. Gene expression and histological analyses demonstrated that, under microgravity conditions, the transition of type I (oxidative) muscle fibres to type IIa (glycolytic) was accelerated in *Nrf2*-KO mice without affecting skeletal muscle mass. Therefore, our results suggest that NRF2 affects myofibre type transition during space flight.

## Abstract (web summary)

Hayashi et al. report on the effect of space flight on muscle mass, fibre size and type, composition and gene signalling of muscle in WT and *Nrf2*-KO mice. Their results demonstrate a key role of NRF2 in maintaining the slow-type fibres of soleus muscle during space flight and in microgravity environments.

## Introduction

Loss of skeletal muscle is caused by a variety of factors, such as cancer cachexia, diabetes, amyotrophic lateral sclerosis (ALS) and long-term inactivity, including that associated with space flight (FL) and ageing, namely sarcopenia^1,2^. Skeletal muscle atrophy is associated with muscle weakness and reduced mobility, resulting in a substantial loss of quality of life. In addition, skeletal muscle, which represents the largest metabolic and secretory organ in the human body, participates in crosstalk with other tissues, such as the liver, bone and brain. Therefore, long-term muscle wasting and dysfunction of skeletal muscles have been linked to the onset of diabetes, cardiovascular diseases, and an increase in mortality^3,4^.

Skeletal muscle mass is tightly regulated by a well-balanced rate of protein synthesis and degradation^5^. In atrophic muscles resulting from disuse, the homoeostatic balance between protein synthesis and degradation (proteostasis) becomes disrupted, favouring increased degradation and reduced synthesis. Proteostasis is primarily regulated by the protein kinase B (AKT)/mammalian target of rapamycin (mTOR) signalling pathway, as well as the ubiquitin–proteasome, and autophagy–lysosome systems^6^. However, mTOR represents one of the most established contributors to the regulation of skeletal muscle mass. mTOR complexes are essential for a variety of cellular processes, including protein synthesis, mitochondrial function, and energy production^7^. Hence, mTOR signalling has been shown to be indispensable for skeletal muscle homoeostasis. Nevertheless, the ubiquitin–proteasome system and autophagy system are two major protein degradation pathways, both of which are associated with muscle atrophy by inducing degradation of muscle structural proteins as well as organelles through enzymatic and lysosome-mediated reactions, respectively. The muscle atrophy-related genes, designated atrogenes, regulate skeletal muscle mass. Specifically, two genes encoding the E3 ubiquitin ligase, muscle RING finger 1 (*MuRF1; Trim63*), and muscle atrophy F-box (*MAFbx; Fbxo32*) represent major atrogenes. Indeed, denervation-induced muscle atrophy is significantly inhibited by deletion of *MuRF1* or *MAFbx*^8^. Meanwhile, *MuRF1* was recently reported to be dispensable for microgravity-induced muscle atrophy in space^9^, suggesting that microgravity-induced muscle atrophy is caused by a mechanism that differs from that of other atrophic conditions on Earth, such as sarcopenia, cancer cachexia, and disuse. In addition, forkhead box-containing protein O1 (*Foxo1*) regulates a broad range of atrophy-related genes, including *MuRF1* and *Fbxo32*^10^. In fact, muscle-specific *Foxo1*-KO mice have been shown to exhibit reduced numbers of slow muscle fibres^11^. Hence, deciphering the molecular mechanisms underlying these anabolic and catabolic pathways involved in skeletal muscle adaptation in various environments could help to prevent muscle wasting conditions, such as sarcopenia, cancer cachexia and microgravity during FL.

In addition to morphological adaptations caused by disuse, skeletal muscles also remodel their metabolic profile and transition fibre types to allow for metabolic adaptation in an atrophic environment. Specifically, disuse, sciatic nerve denervation, tail suspension and microgravity have been shown to stimulate slow-to-fast fibre transition^12–14^, whereas fasting and ageing induce fast-to-slow fibre transition^15–17^. However, the molecular mechanisms associated with skeletal muscle atrophy and the myofibre type transition remain unclear, with no effective treatment or prevention having been established.

Several studies have been conducted to investigate the effects of microgravity during FL on skeletal muscle homoeostasis in humans, rats, and mice^13,18,19^. However, these studies were unable to exclude the possibility that other factors in the space environment, such as space radiation, shock related to launching, or mental stress, influenced the disruption of skeletal muscle homoeostasis. To investigate the direct effect of microgravity on skeletal muscle, the Japan Aerospace Exploration Agency (JAXA) developed the multiple artificial gravity research system ‘MARS’ that can simulate artificial gravity 1 *g* in space using a centrifuge^20–22^. Using this system, we have previously shown that the artificial gravity of 1 *g* in space is comparable to ground control (GC) regarding skeletal muscle mass^21^. In the space environment, type I fibre-dominant muscles, such as the soleus muscles, are more sensitive to microgravity than type II fibre-dominant muscles, resulting in a severe loss of muscle mass and slow-to-fast fibre transition^23^.

Redox status regulates a variety of cellular functions. Excessive reactive oxygen species (ROS) production disrupts the redox status, leading to oxidative stress^24^, which is related to the regulation of skeletal muscle plasticity, including atrophy and hypertrophy, as well as muscle fibre type transition^25,26^. Meanwhile, nuclear factor (erythroid derived 2)-like 2 (NRF2) is a master transcription factor that regulates defence mechanisms against oxidative stress^27^. In our previous report, NRF2 activity was induced during FL in many tissues including the thymus and white adipocytes^28^.

In the current study, we used *Nrf2*-knockout (KO) mice to study the direct function of NRF2 in soleus muscle adaptation during FL. We adopted the Mouse Habitat Unit (MHU) to launch and manage genetically engineered animals with advanced technology in space^21^. Results show that the soleus muscle mass, as well as the size of each fibre type, decreased at a rate that was almost identical between wild-type (WT) and KO mice after FL. However, interestingly, RNA sequencing (RNA-seq) and immunohistochemical analyses demonstrated that KO mice exhibited an acceleration of type I to type IIa transition after FL. Here, we propose a function for NRF2 in skeletal muscle by which it affects the slow-to-fast fibre type transition during skeletal muscle disuse in space by regulating oxidative and metabolic responses.

## Results

### *Nrf2* deletion does not accelerate nor prevent microgravity-induced skeletal muscle atrophy

**Fig. 1:**
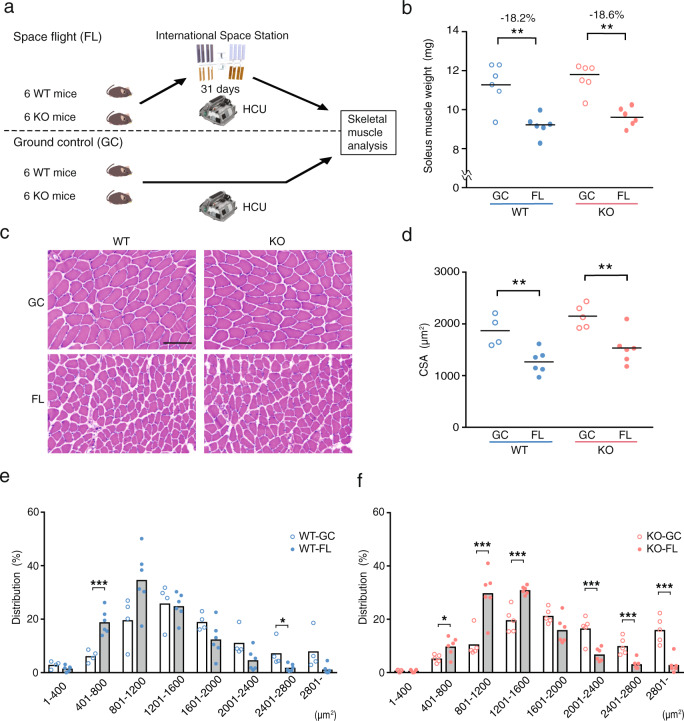
Impact of *Nrf2* deficiency on the soleus muscles of mice in a space experiment. **a** Overview of the space experiment (MHU-3 mission). Six WT and six KO mice were launched in the Japanese Experiment Module ‘KIBO’ on the ISS by SpX14 from the Kennedy Space Center in Florida. They were housed in ‘KIBO’ for 31 days and were then returned to Earth. **b** Absolute soleus muscle weights from WT-GC (*n* = 6), WT-FL (*n* = 6), KO-GC (*n* = 6) and KO-FL (*n* = 6) mice. **c** Haematoxylin–eosin staining of soleus muscle fibre sections. Scale bar = 100 μm. **d** CSA of each fibre type in WT-GC (*n* = 6), WT-FL (*n* = 4), KO-GC (*n* = 5) and KO-FL (*n* = 6) soleus muscles. **e** Fibre area frequency distribution in WT-GC vs. WT-FL. **f** Fibre area frequency distribution in KO-GC vs. KO-FL. *P* values calculated using Student’s *t* test are indicated as follows: *P < 0.05, **P < 0.01, ***P < 0.001, GC vs. FL.

Six WT mice and six KO mice were launched into the Japanese Experiment Module ‘KIBO’ of the International Space Station (ISS) and housed for 31 days in the Habitat Cage Unit (HCU) (FL mice). The FL mice were dissected 2 days after their return to Earth. A GC experiment was conducted to simulate the FL experiment (GC mice; Fig. 1a). Moreover, considering our recent findings that in the FL groups, body weight gain in KO (102.47%) mice is significantly lesser than that of WT (107.85%) mice^28^, we sought to elucidate the effect of NRF2 on skeletal muscle mass during FL. To this end, we measured the wet weight of the hindlimb skeletal muscles: soleus, plantaris, gastrocnemius, tibialis anterior and extensor digitorum longus (EDL) (Fig. 1b and Supplementary Fig. 1a). The weight of the soleus muscle, an antigravity muscle that is reportedly susceptible to microgravity^29–31^, exhibited significant atrophy in both WT-FL and KO-FL after FL (Fig. 1b). The rate of change was nearly identical between both groups (Supplementary Fig. 1b). Meanwhile, skeletal muscle weight normalised by body weight and rate of change appeared to differ between WT and KO mice (Supplementary Fig. 2a, b). This discrepancy can be explained by the lower weight gain rate in KO mice during FL^28^. Furthermore, haematoxylin–eosin staining of soleus and EDL muscle cross-sections in WT and KO mice showed no evidence of central nucleation or immune cell infiltration, which are hallmarks of skeletal muscle abnormalities (Fig. 1c and Supplementary Fig. 3a). Cross-sectional area (CSA) and distribution analysis of soleus muscle further demonstrated a reduction in fibre size in both FL mice (Fig. 1d–f). In addition, two-way analysis of variance (ANOVA) showed significant difference in the genotypic factor of CSA (Supplementary Table 1), whereas the EDL muscle, which is mainly composed of fast-twitch fibres, did not exhibit reduction in CSA, and distribution analysis did not exhibit major changes in both WT and KO after FL (Supplementary Fig. 3b–d). Number of analysed fibres are shown in Supplementary Table 2. These results suggest that NRF2 is not involved in the maintenance of skeletal muscle mass during prolonged exposure to a microgravity environment.

### Gene expression variations are similar between WT and *Nrf2*-KO mice under microgravity

**Fig. 2:**
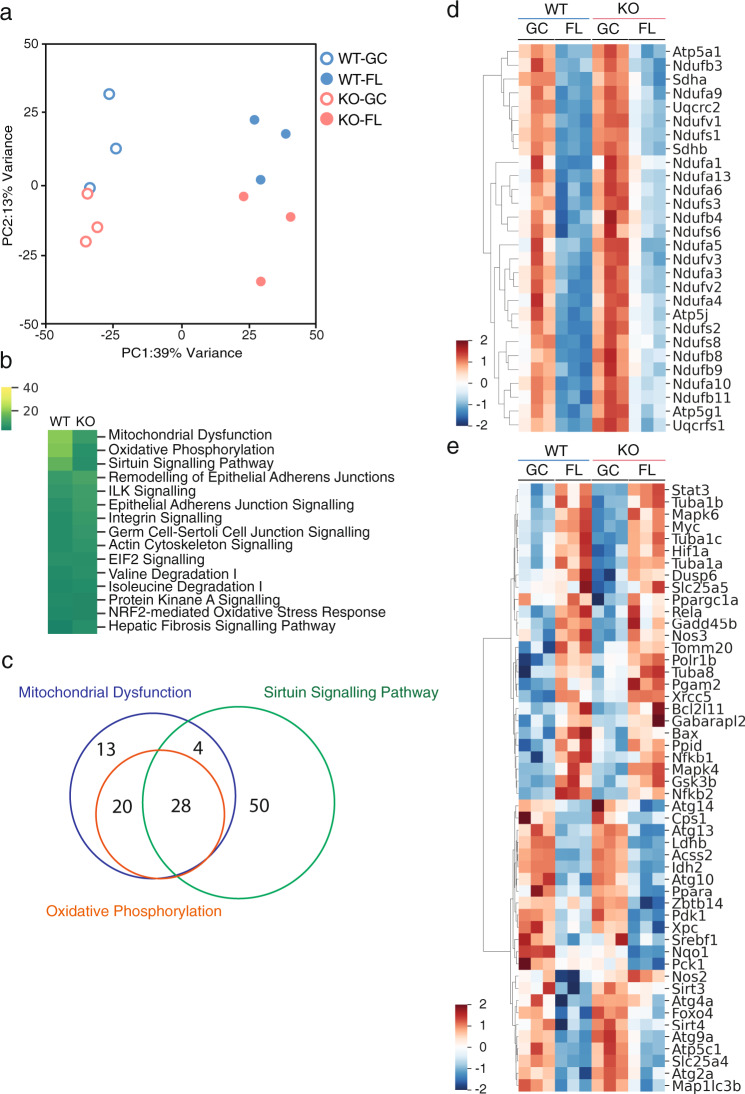
Comprehensive analysis of gene expression by RNA sequence and pathway analysis. **a** PCA plot of overall gene expression in at least one comparison among WT-GC (*n* = 3), WT-FL (*n* = 3), KO-GC (*n* = 3) and KO-FL (*n* = 3) soleus muscles. **b** Top 15 pathway analysis of differentially expressed genes (DEGs) in WT (GC vs. FL) and KO (GC vs. FL). **c** Venn diagram of DEGs associated with each of the Mitochondrial Dysfunction, Oxidative Phosphorylation, and Sirtuin Signalling Pathway. **d** Heatmap of the expression of 28 genes overlapping in the three pathways. **e** Heatmap of the expression of 50 genes specific to the Sirtuin Signalling Pathway.

To investigate whether NRF2 deficiency affects global gene expression patterns during FL, we performed RNA-seq analysis on soleus muscles. Although slight gene variabilities were observed between WT and KO mice before FL, overall gene expression after FL was similarly altered in both WT and KO mice based on a principal component analysis (PCA) of all genes (Fig. 2a). A total of 1130 differentially expressed genes (DEGs) were enriched by ANOVA with a false discovery rate (FDR) < 0.05. Moreover, a heatmap showed that the same variability was observed in both groups (WT, KO) under each environment (GC, FL); however, certain gene expression patterns were found to differ between WT and KO mice (Supplementary Fig. 4a). Specifically, in the FL groups, 962 genes were upregulated and 1004 genes were downregulated, while a total of 1966 genes were differentially regulated in WT mice compared to GC mice (Supplementary Fig. 4b). Meanwhile, 1159 genes were upregulated and 1124 were downregulated in the KO mice, for a total of 2283 genes (Supplementary Fig. 4c) in the edgeR test FDR < 0.05.

To investigate how each DEG functions, we performed Ingenuity Pathway Analysis (IPA). The top 15 pathways based on *P* values that are highly associated with the variant genes are shown. The affected pathways in both WT and *Nrf2*-KO mice were almost identical (Fig. 2b). Comparison between WT and KO among 15 pathways suggests that most pathways are regulated to the same extent. To compare the genes, we focused on the top three pathways: Mitochondrial Dysfunction, Oxidative Phosphorylation and Sirtuin Signalling. Genes related to each pathway are shown by a Venn diagram (Fig. 2c). Twenty-eight genes are common in the three pathways. A heatmap of the expression of the 28 genes exhibited the same tendency between WT and KO after FL, although the degree of variation seemed to differ (Fig. 2d). However, in the heatmap of the 50 genes specific to the Sirtuin Signalling Pathway, although most genes showed a similar tendency, the expression patterns of *Pck1* and *Nos2* were differentially affected by FL in WT and KO (Fig. 2e). Although some of the specific pathways associated with skeletal muscle plasticity were altered by NRF2 deletion under microgravity conditions, the overall gene variations between WT and KO mice were similar after FL.

### Deletion of *Nrf2* does not affect atrogene transcription in microgravity

**Fig. 3:**
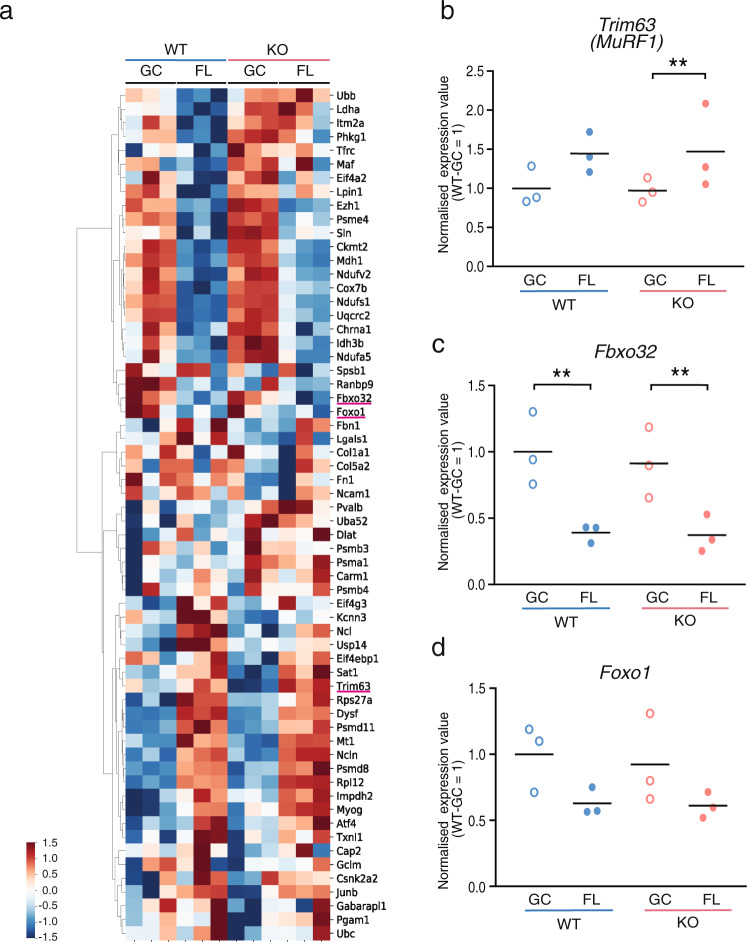
Atrogene expression in space experiment. **a** Heatmap of atrogene expression values in soleus muscles from WT-GC, WT-FL, KO-GC and KO-FL mice. Expression values of *Trim63* (*MuRF1*) (**b**), *Fbxo32* (**c**) and *Foxo1* (**d**) genes in soleus muscles. Data are presented as mean, and each point represents individual mice. *n* = 3 for WT-GC, WT-FL, KO-GC and KO-FL. edgeR test **P < 0.01 (FDR-corrected).

Skeletal muscle atrophy is generally regulated by transcriptional activation of genes called atrogenes^8,32^. Atrogenes are upregulated or downregulated in fasting, cancer cachexia, uraemia, diabetes mellitus, disuse and denervation^33,34^. Next, we investigated whether the *Nrf2* deletion influences various atrogenes at the transcriptional level. A heatmap showed that most atrogenes in the WT and KO mice had a similar expression trend (Fig. 3a), including *MuRF1*, *Fbxo32* and *Foxo1* (Fig. 3b–d). These results suggest that the loss of NRF2 activity does not alter the transcription of atrogenes in FL-induced atrophy.

### Expression of NRF2-target genes is more significantly suppressed under microgravity in KO mice compared to WT

**Fig. 4:**
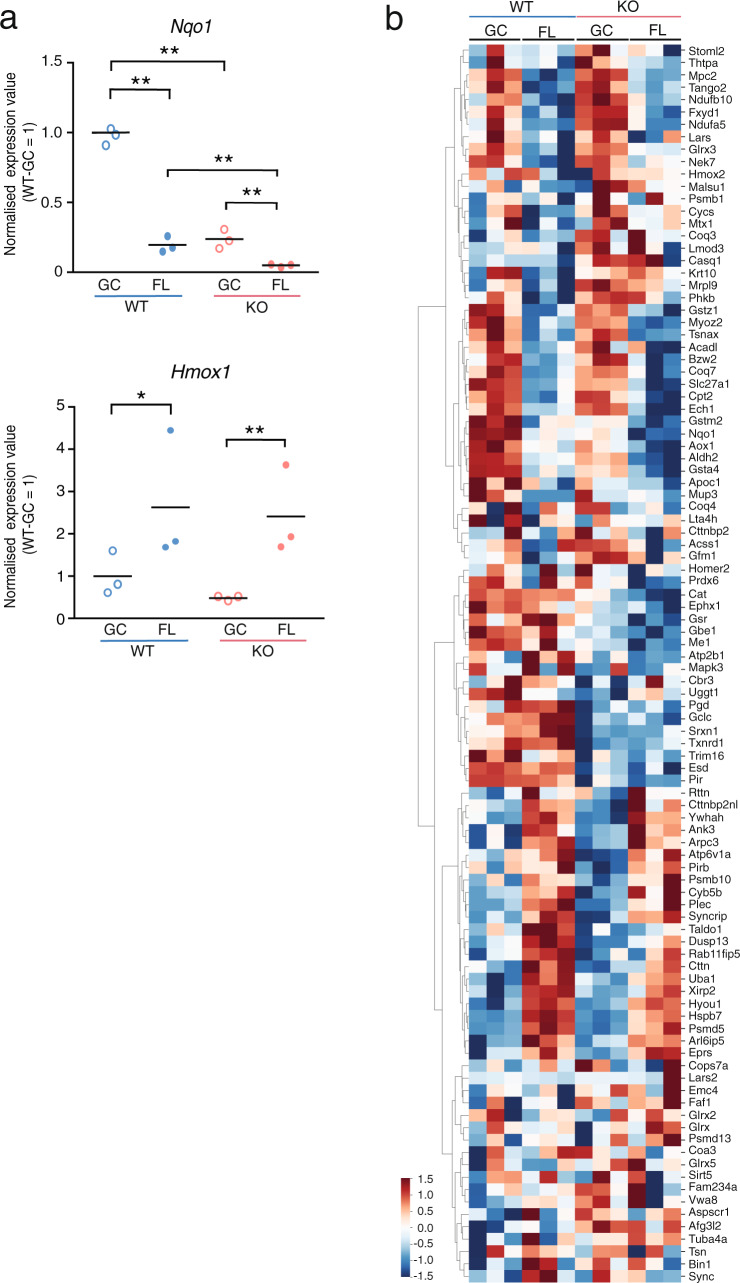
Pattern of NRF2-target gene expression in soleus muscles. **a** Expression of *Hmox1* and *Nqo1* genes in soleus muscles. *n* = 3 for WT-GC, WT-FL, KO-GC and KO-FL. edgeR test *P < 0.05, **P < 0.01 (FDR-corrected). **b** Heatmap depicting the gene expression patterns of upregulated genes in skeletal muscle-specific *Keap1-*knockout mice.

We next focused on well-known NRF2 regulated genes in skeletal muscle. In both WT and KO mice, the expression of *Nqo1* was greatly reduced in FL, however, the extent of reduction was greater in KO mice (Fig. 4a). Meanwhile, *Hmox1* expression was significantly upregulated to the same extent in both WT and KO mice under FL conditions compared to GC conditions, however, its expression tended to lower in KO mice than WT mice (Fig. 4a). These results indicate that *Hmox1* is induced in an NRF2-independent manner under FL conditions. In addition, a heatmap was prepared for genes with increased protein expression in skeletal muscle-specific *Keap1*-KO mice^35^, which are suggested to be regulated by Nrf2 in skeletal muscles. The data showed no difference between WT and KO regarding changes in gene expression owing to FL (Fig. 4b). Taken together, these gene expression patterns suggest that minimal induction of NRF2 downstream gene expression occurs in soleus muscle under FL conditions.

### *Nrf2*-KO mice accelerate slow-to-fast fibre type transition in microgravity

**Fig. 5:**
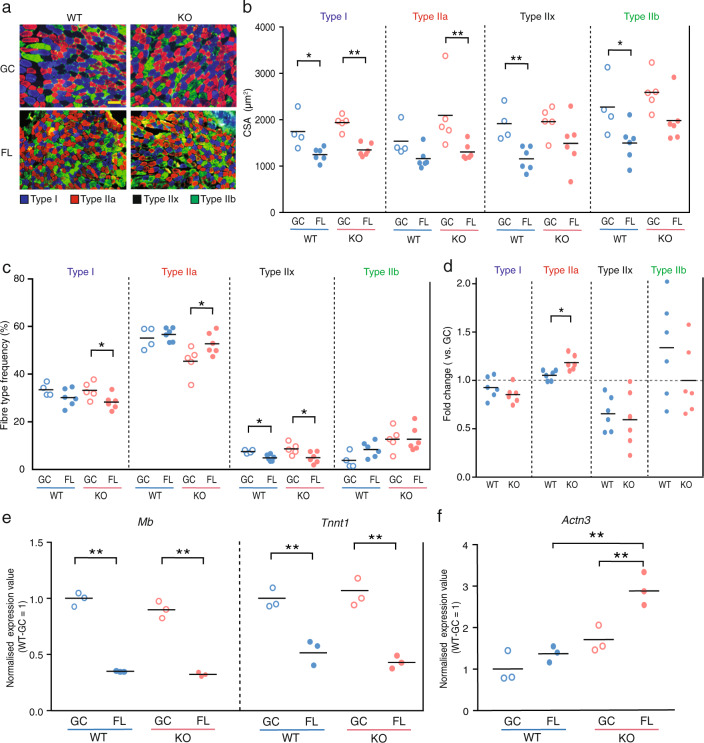
Effect of *Nrf2* deficiency on fibre type transition. **a** Immunohistochemical staining for myosin heavy chain using BA-D5 (type I; blue), SC-71 (type IIa; red), and BF-F3 (type IIb; green) antibodies. Unstained fibres predicted to be type IIx (black). Scale bar = 100 μm. **b** CSA of each fibre type from WT-GC (*n* = 6), WT-FL (*n* = 6), KO-GC (*n* = 6) and KO-FL (*n* = 6). **c** Frequency of each fibre type in WT-GC (*n* = 6), WT-FL (*n* = 6), KO-GC (*n* = 6) and KO-FL (*n* = 6) mice. **d** Comparison of FL fibre type frequency normalised by GC fibre type frequency from WT and KO mice. *P* values from Student’s *t* tests (**b**–**d**) are indicated as follows: *P < 0.05, **P < 0.01, GC vs. FL. *n* = 6 for WT-FL and KO-FL, *n* = 5 for KO-GC and *n* = 4 for WT-GC. **e** Expression of *Mb* and *Tnnt1* in soleus muscles. **f** Expression value of *Actn3* in soleus muscles. Expression values were normalised by Quantile (WT-GC = 1). *n* = 3 for WT-GC, WT-FL, KO-GC and KO-FL. edgeR test (**e**, **f**) **P < 0.01 (FDR-corrected).

Oxidative metabolism is primarily carried out in slow (type I) muscle fibres, whereas glycolytic metabolism occurs in fast (type II) muscle fibres. The soleus muscles under a microgravity environment undergoes a transition from slow-to-fast muscle fibres^23,36^. Therefore, we next investigated the effect of KO mice on the fibre type transition after FL. The soleus and the EDL muscle cross-sections were immunostained with specific antibodies against type I, IIa and IIb muscle fibres. Neither type I, type IIa, nor type IIb were defined as type IIx (Fig. 5a and Supplementary Fig. 5a). In all WT and KO fibre types, significant decreases were observed in the muscle fibre size of FL mice compared to that in GC mice in soleus muscle (Fig. 5b). These results suggest that the size of all fibre types in the soleus muscles during microgravity-induced atrophy may be regulated by mechanisms independent of NRF2, which agrees with the findings presented in Fig. 1.

Next, we analysed the ratio of each fibre type after FL in both WT and KO mice. Intriguingly, the ratio of type I fibres in KO mice was significantly decreased by the microgravity environment, while this effect was not detected in WT (Fig. 5c, d). Furthermore, the ratio of type IIa fibres in KO mice was significantly increased by the microgravity environment, which was not observed in WT mice (Fig. 5c, d). The EDL muscle, a fast-type muscle, that did not undergo atrophy after FL, exhibited an increase in type IIa fibres and a decrease in type IIb fibres in both WT-FL and KO-FL (Supplementary Fig. 5b). However, the rate of change was almost identical between WT and KO (Supplementary Fig. 5c).

To further confirm the fibre type transition in soleus muscle in KO mice after FL, we analysed the slow fibre-related genes myoglobin (*Mb*), troponin T1 (*Tnnt1*) and fast fibre-related gene actinin3 (*Actn3*)^37,38^. Although *Mb* and *Tnnt1* were downregulated in both WT-FL and KO-FL mice (Fig. 5e), *Actn3* was significantly upregulated only in KO mice after FL (Fig. 5f). These results suggest that NRF2 may affect the fibre type transition rather than the soleus muscle fibre size during FL.

### NRF2 deficiency in microgravity conditions induces the expression of glycolytic metabolism-related genes

**Fig. 6:**
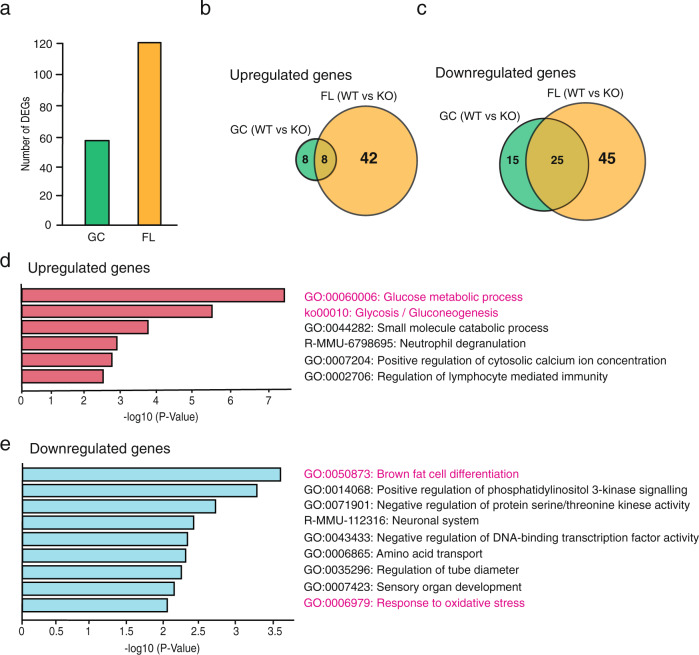
Enrichment analysis of differentially expressed genes between WT and KO mice. **a** The number of DEGs identified in GC (WT vs. KO) and FL (WT vs. KO) mice. Venn diagram depicting the comparison of upregulated genes (**b**) or downregulated genes (**c**) in WT vs. KO mice under GC and FL conditions. Enrichment analysis of KO-FL-specific DEGs including 42 upregulated genes (**d**), and 45 downregulated genes (**e**).

Since the ratio of fibre types was altered in KO mice after FL, we sought to more closely examine the DEGs among the genotypes (WT vs. KO) and gravity conditions (GC vs. FL). Only 56 DEGs were detected between WT-GC and KO-GC mice (Fig. 6a), of which 16 were upregulated, and 40 were downregulated by *Nrf2* deletion. In contrast, 120 DEGs were identified between WT-FL and KO-FL mice, of which 50 genes were upregulated, and 70 were downregulated by *Nrf2* deletion.

Next, to determine which gene variations were specifically altered by the combination of FL and *Nrf2* deletion, DEGs that were simultaneously identified in FL mice and DEGs in GC mice were removed, resulting in 42 upregulated and 45 downregulated genes (Fig. 6b, c). The pathway analysis for these 42 genes revealed that the upregulated genes were related to glucose metabolic processes and glycolysis/glycogenesis, suggesting that glycolytic metabolism was enhanced in KO mice (Fig. 6d). Meanwhile, brown fat cell differentiation was determined to be the most significantly altered pathway associated with the 45 downregulated genes (Fig. 6e). Together, these results support that *Nrf2* deletion accelerates the transition from slow oxidative to fast glycolytic processes in soleus muscles during FL.

## Discussion

In the present study, we investigated the role of NRF2 in skeletal muscle during microgravity-induced atrophy in space. Since soleus muscles are predominantly composed of slow-type fibres, they contain a significantly higher level of NRF2 protein as well as other oxidative stress-related molecules, such as NQO1 and SOD (superoxide dismutase) compared to white vastus muscles that are primarily composed of fast-type fibres^39^. Thus, considering that soleus muscles are more sensitive to microgravity, NRF2 may be a potent regulator of antioxidative stress response during skeletal muscle disuse in space. Since NRF2 is a master regulator of antioxidant response and we previously found its activity to be upregulated in response to microgravity-induced oxidative stress in all tissues^28^, we hypothesised that the deletion of *Nrf2* would accelerate skeletal muscle atrophy during FL. However, we unexpectedly found that *Nrf2* deletion influenced the fibre type transition in skeletal muscles during microgravity-induced atrophy, without affecting the size or weight of skeletal muscles. Specifically, KO mice with prolonged exposure to a microgravity environment exhibited reduced type I fibres and enhanced type IIa fibres in soleus muscle. As for the EDL muscle, which is predominantly composed of fast-twitch fibres, weight loss was observed in KO-FL, but no significant decrease in CSA was observed. This suggests that FL did not cause muscle atrophy in EDL muscle, nor was it accelerated by the deficiency of *Nrf2*. In addition, we found an increase in type IIa and a decrease in type IIb, which may be a compensatory change for the fast-twitch soleus muscle. However, there was no significant difference in the rate of change between WT and KO, suggesting that the deficiency of *Nrf2* has little effect on EDL muscle. Therefore, the present study focused on soleus muscle, which is more susceptible to *Nrf2* deficiency.

Consistent with the fibre type transition, a pathway analysis based on RNA-seq data revealed that oxidative response was downregulated while glycolytic metabolism, as well as expression of *Actn3* in fast-type fibres, were enriched in soleus muscle of NRF2-deficient mice after FL.

ROS and oxidative stress regulate a variety of cellular processes, including protein turnover. In skeletal muscles, a number of previous studies have reported that prolonged muscle inactivity leads to increased production of ROS, which in turn induces proteolysis and suppressed protein synthesis. In addition, evidence suggests that NRF2 plays a role in regulating skeletal muscle mass in various atrophy models, including ageing^40^, cancer cachexia^41^, ALS^42^ and disuse^43,44^. Nonetheless, whether ROS and oxidative stress are major factors contributing to microgravity-induced atrophy remains controversial. After FL, there was no difference in the weight of muscles except the EDL muscle between WT and KO mice (Fig. 1b and Supplementary Figs. 1, 2). However, the CSA of the EDL muscle did not decrease after FL (Supplementary Fig. 3b). We found that among the *Sod* genes (*Sod1*, *2* and *3*) in the soleus muscles, only *Sod2* (mitochondrial SOD) was downregulated after FL (Supplementary Fig. 6a). Consistent with this, the levels of Nqo1 antioxidant enzyme also decreased in soleus muscles after FL, though the reason is not clear. One possibility is that the prolonged inactivity-induced atrophy causes the downregulation of antioxidant capacity^6^. We collected the tissue samples 2 days after arrival on Earth; therefore, short-term exposure of FL mice to 1 *g* during the return might have affected the gene expression.

The expression of major atrogenes, *MuRF1* and *Fbxo32*, was not altered by FL with the deletion of *Nrf2* (Fig. 3b, c). Moreover, we had previously shown that NRF2 activity is significantly enhanced in other tissues^28^. However, in the present study, we did not observe NRF2 activation in soleus muscles, which mostly comprises slow oxidative fibres (Fig. 4). In fact, the activity of NRF2 was reduced in skeletal muscle under a microgravity environment, thus suggesting that the space environment may suppress NRF2 activity in skeletal muscles. In addition, KO caused more severe suppression of its target genes (Fig. 4). One of the possible mechanisms of NRF2 repression in soleus after FL is increased phosphorylation of NRF2 by GSK-3β^45^. We checked *Ppargc1a* (*PGC-1α*) mRNA level because it is negatively regulated by GSK-3β^46^. We found no significant difference of *Ppargc1a* expression between GC and FL in WT (Supplementary Fig. 6b). Another possible mechanism is increased expression of *Keap1*^47^, but there was no difference in the expression of this gene either (Supplementary Fig. 6b), suggesting that NRF2 is negatively affected by an as yet unknown mechanism.

Collectively, NRF2 may be dispensable for microgravity-induced muscle atrophy in space, whereas targeting NRF2 or NRF2-dependent pathways could be effective in other types of atrophy models on Earth.

In general, skeletal muscle atrophy caused by disuse, denervation and FL induces fibre atrophy with a slow-to-fast-type transition, whereas ageing and cancer cachexia preferentially affect type 2 fibre with a fast-to-slow-type shift^48^. Intriguingly, we found that NRF2-deficient mice had a lower proportion of type I fibres with a corresponding higher proportion of type II fibres following prolonged microgravity exposure, compared to WT. We also successfully obtained data supporting the type I to type IIa fibre transition in KO mice, based on RNA-seq analysis (Fig. 6d). Although whole gene expression analysis indicated that most genes were similarly differentially expressed after FL in WT and KO mice, there were some exceptions that may be associated with fibre type transition (Supplementary Figs. 4a and 5f). Specifically, the pathway analysis clearly suggests that oxidative response was downregulated while glycolytic metabolism and the glycolysis pathway were enriched in KO mice after FL, suggesting that oxidative (slow)-to-glycolytic (fast) fibre transition was preferentially induced in the absence of NRF2 activity. This result is consistent with a previous report demonstrating that treatment with Trolox, an antioxidant, in mice experiencing disuse skeletal muscle atrophy did not prevent skeletal muscle atrophy, however, did prevent slow-to-fast fibre transition^25^. In addition, a previous study reported that KO mice displayed shorter running distance during forced treadmill exercise compared to WT mice, while treatment with an NRF2 activator enhanced running distance^49^. Taken together, our results suggest that oxidative stress could contribute to the transition of skeletal fibre types in the microgravity space environments. Thus, targeting NRF2 may prove beneficial for protecting against fibre transition during FL.

According to IPA analysis of the DEGs affected by the microgravity environment, Mitochondrial Dysfunction, Oxidative Phosphorylation and Sirtuin Signalling Pathway had 28 genes showing a similar pattern of change, indicating that the main gene expression changes were not altered by *Nrf2* deficiency. However, from the heatmap of Sirtuin signalling pathway-specific genes, *Pck1* and *Nos2* seemed to exhibit different patterns between WT and KO. PCK1 is a promoter of glycogenesis, an important factor in metabolic changes^50^, and NOS2 is nitric oxide synthase 2, which produces nitric oxide (NO). It has been reported that NO signalling is regulated by skeletal muscle contraction and metabolic responses^51^. Further analysis is required to address whether this finding is related to the enhancement of fast-twitch muscle growth by *Nrf2* deficiency. However, it remains unclear whether NRF2 directly regulates these pathways to mediate fibre type transition in a microgravity environment. Therefore, it will be of interest to investigate the NRF2-target pathway responsible for regulating skeletal muscle plasticity, particularly relating to fibre type switching, during FL, in future studies.

Certain inherent disadvantages and advantages are associated with performing space studies. Our present study strongly suggests that to understand the molecular mechanisms underlying the induction of skeletal muscle atrophy in space, it is essential to perform animal experiments in space, since the space environment is unique. Meanwhile, since our samples were collected 2 days after the animals returned to Earth, we may have observed adaptation responses. Nevertheless, this is the first report demonstrating the function of NRF2 in skeletal muscles during a prolonged period of exposure to a microgravity environment.

Here, we show that the deletion of NRF2 did not affect skeletal muscle mass after FL, however, we unexpectedly discovered a potentially function for NRF2 related to fibre type transition during FL.

## Materials and methods

### The MHU-3 mission

The HCU equipped with a food and watering system was used for the MHU-3 project. Six WT mice and six *Nrf2*-KO mice (11 weeks old) were launched in the SpaceX Falcon 14 rocket with transportation cage units (TCU) into the Japanese Experiment Module ‘KIBO’ of the ISS. They were housed for 31 days in the FL group, which was also equipped with a camera to monitor each mouse’s behaviour. After 31 days, the mice were loaded into the Dragon capsule and returned to the Pacific Ocean, offshore from Southern California, on May 5. All mice were retrieved by ship 2 days after landing in the Pacific Ocean, and sent to Explora BioLabs. A GC experiment to simulate a space experiment was then conducted at JAXA in Tsukuba, Japan, from September 17 to October 20, 2018.

During the flight mission, a temperature/humidity data logger was attached to the TCU during the launch, the on-board and the return phases, and inside the Cell Biology Experiment Facility during the on-board phase. Data from the logger were processed after the mission. Average temperatures during the launch, the on-board and the return phases were 23.8, 22.9 and 23.7 °C, respectively. Average temperature during dissection in the Explora Biolabs was 21.2 °C. The average relative humidity during the launch, the on-board and the return phases were 43.1%, 52.1%, and 43.9%, respectively. Mice were housed under a 12:12-h light/dark cycle adjusted to GMT from pre-launch acclimation to return phase.

### Mice

*Nrf2*-KO mice (Nfe2l2^tm1Ymk^)^52^ and WT male mice on the C57BL/6J background were bred at Charles River Laboratories Japan for MHU-3. All animal experiments were approved by the Institutional Animal Care and Use Committees of the University of Tsukuba (No. 18-451), JAXA (Protocol Numbers 017-001 and 017-014), National Aeronautics and Space Administration (Protocol Number FLT-17-112) and Explora BioLabs (Study Number: EB15-010C), and conducted according to the related guidelines and applicable laws of Japan and the United States of America. After all FL mice arrived alive at the Explora BioLabs, they were first subjected to health check and body weights measurements. Then, behavioural tests (open field, light/dark transition and Y-maze tests) were conducted before blood collection from the tail. FL mice were anesthetised with isoflurane, euthanised by exsanguination and then dissected for tissue collection. GC mice were treated similarly. The skeletal muscles used in this study were from the same animals that were analysed in a study by Suzuki et al.^28^.

### RNA-seq analysis

Total RNA was extracted from 100 sections of 8-μm frozen soleus muscle tissues using TRIzol reagent (Thermo Fisher Scientific). The RNA-seq library was prepared using the NEBNext Ultra Directional RNA Library Prep Kit (New England Biolabs [NEB]) after rRNA depletion (NEBNext rRNA Depletion Kit; NEB). Paired-end (2 × 36 bases) sequencing was performed using NextSeq500 (Illumina). FASTQ files were imported to the CLC Genomics Workbench (Version 10.1.1; Qiagen). Sequence reads were mapped to the mouse reference genome (mm10). Gene expression levels were calculated as total read counts normalised by the quantile method. Genes with 0 counts in any sample were excluded, and differential expression was analysed using the Empirical Analysis of DGE tool (edgeR test) in CLC Main Workbench (Version 7.7.3; Qiagen). Comparisons between four groups were conducted using one-way ANOVA with Tukey–Kramer test. DEGs were extracted among conditions (WT-GC vs. KO-GC vs. WT-FL vs. KO-FL) with FDR-corrected *P* < 0.05. PCA plots were constructed using iDEP (http://bioinformatics.sdstate.edu/idep/).

### Gene functional analysis process

IPA (Qiagen) was used for the pathway analysis of DEGs for WT (GC vs. FL) or *Nrf2*-KO (GC vs. FL, Fig. 2b). Metascape^53^ (https://metascape.org/gp/index.html#/main/step1) was used for enrichment analysis of specific DEGs in WT vs. *Nrf2*-KO mice under microgravity conditions (Fig. 6d, e).

### Histological analysis and immunohistochemistry of muscle cryosections

The left and right soleus or EDL muscles were neatly stacked on top of each other. The Achilles tendon side was vertically mounted on the tragacanth gum on a cork disc, and quickly frozen in isopentane cooled in liquid nitrogen. Thin frozen muscle sections were subjected to immunohistochemical and RNA-seq analyses.

Frozen tissue sections (8 μm in thickness) were mounted on glass slides and subjected to haematoxylin/eosin staining and immunohistochemical analysis.

Tissue sections were immunostained with mouse monoclonal antibody (BA-D5, 1:50) against myosin heavy chain 7 for type I fibre. The others were incubated with 1:100 dilution of mouse monoclonal antibody (SC-71) against myosin heavy chain 2 for type IIa fibres, and 1:100 dilution of mouse monoclonal antibody (BF-F8) against myosin heavy chain 4 for type IIb fibres. Primary antibodies were purchased from the Developmental Studies Hybridoma Bank (University of Iowa). All tissues were stained using a fluorescein M.O.M. kit (Vector Laboratories) in a humid chamber. The sections were air-dried, fixed with −20 °C acetone, washed with PBS, 1% BSA/PBS incubated with the primary antibodies, and M.O.M. protein concentrate after blocking with 5% goat serum/1% BSA/PBS. M.O.M. blocking reagent was the added and incubated overnight at 4 °C. All immunostaining samples were visualised using appropriate species-specific secondary antibodies (Thermo Fisher Scientific) with a 1:500 dilution of Alexa Fluor 350 conjugated anti-mouse IgG2b, 1:1000 dilution of Alexa Fluor 488 conjugated anti-mouse IgG1 and 1:1000 dilution of Alexa Fluor 555 conjugated anti-mouse IgM for 1 h at room temperature. Tissue sections were mounted with VECTASHIELD Vibrance Antifade Mounting Medium (Vector Laboratories). The proportion of each fibre type and the CSA of muscle fibres was assessed in the stained images using a BIOREVO BZ-X800 microscope system and hybrid cell count application (Keyence). All of fibres were counted in soleus and EDL muscles, which contains 443–1585 fibres/section.

### Statistics and reproducibility

All data are presented as the mean with each point representing a biological replicate. The comparison between GC and FL of WT or KO mice was determined using Student’s *t* test, and a *P* < 0.05 with the two-side test was considered significant (Figs. 1b, d and 5b–d). Two-way ANOVA was used for the comparison about soleus muscle wet weight and CSA between GC and FL of WT or KO (Supplementary Table 1). Comparisons between all groups in Fig. 2b were conducted using one-way ANOVA with the Tukey–Kramer test.

### Reporting summary

Further information on research design is available in the Nature Research Reporting Summary linked to this article.

## Supplementary information

Supplementary Information

Description of Additional Supplementary Files

Supplementary Data 1

Supplementary Data 2

Supplementary Data 3

Reporting Summary

## Data Availability

All data that support the findings of this study are available from the corresponding author upon reasonable request. RNA-seq data were deposited in the DDBJ database (The DNA Databank of Japan, https://www.ddbj.nig.ac.jp/) (accession number DRA011091). Source data for graphs presented in the main and Supplementary Figures are provided in Supplementary Data 1–3.
