# Supplementary material for: Nuclear factor E2-related factor 2 (NRF2) deficiency accelerates fast fibre type transition in soleus muscle during space flight
Source: Commun Biol. 2021 Jun 24;4:787. doi: 10.1038/s42003-021-02334-4 (PMC8225765; doi:10.1038/s42003-021-02334-4)
Supplement: Supplementary file 1 — Supplementary Information [file 42003_2021_2334_MOESM1_ESM.pdf]

Nuclear factor E2-related factor 2 (NRF2) deficiency accelerates fast fibre type transition in soleus muscle during space flight

Takuto Hayashi[1,2)], Takashi Kudo[1*)], Ryo Fujita[3)], Shin-ichiro Fujita[2,4)], Hirona Tsubouchi[1)], Sayaka Fuseya[1,2)], Riku Suzuki[1,5)], Michito Hamada[1)], Risa Okada[6)], Masafumi Muratani[4)], Dai Shiba[6)], Takafumi Suzuki[7)], Eiji Warabi[1*)], Masayuki Yamamoto[7)], Satoru Takahashi[1*)]

[1]Laboratory Animal Resource Center in Transborder Medical Research Center, and Department of Anatomy and Embryology, Faculty of Medicine, University of Tsukuba, Ibaraki 305-8575, Japan
[2]Doctoral Program in Biomedical Sciences, Graduate School of Comprehensive Human Sciences, University of Tsukuba, Ibaraki 305-8575, Japan
[3]Divsion of Regenerative Medicine, Transborder Medical Research Center, Faculty of Medicine, University of Tsukuba Ibaraki 305-8575, Japan.
[4]Department of Genome Biology, Transborder Medical Research Center, Faculty of Medicine, University of Tsukuba, Ibaraki 305-8575, Japan.
[5]Ph.D. Program in Human Biology, School of Integrative and Global Majors, University of Tsukuba, Ibaraki 305-8575, Japan.
[6]JEM Utilization Center, Human Spaceflight Technology Directorate, JAXA, Ibaraki 305-8505, Japan
[7]Department of Medical Biochemistry, Tohoku University Graduate School of Medicine, Sendai 980-8575, Japan

***Corresponding authors:**
Eiji Warabi, Ph.D., E-mail: warabi-e@md.tsukuba.ac.jp
Takashi Kudo, Ph.D., E-mail: t-kudo@md.tsukuba.ac.jp
Satoru Takahashi, M.D., Ph.D., E-mail: satoruta@md.tsukuba.ac.jp
Department of Anatomy and Embryology, Faculty of Medicine, University of Tsukuba, 1-1-1 Tennodai, Tsukuba, Ibaraki, 305-8575, Japan.
Tel: +81-298-53-7516; Fax: +81-298-53-6965;

Supplementary Figure 1

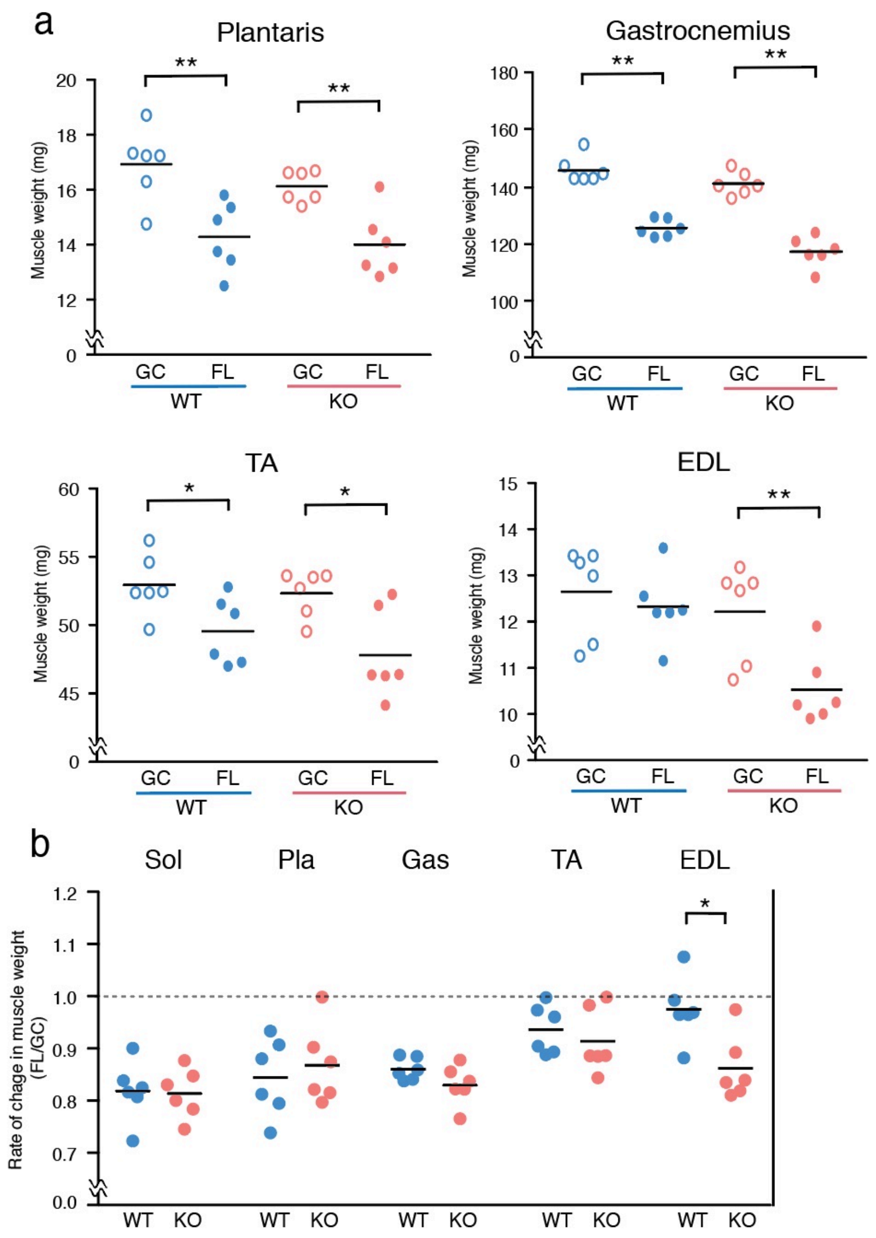

Supplementary Figure 1. Absolute skeletal muscle weight in space flight experiment

(a) Absolute skeletal muscle weights from WT-FL (n = 6), WT-GC (n = 6), KO-GC (n = 6), and KO-FL (n = 6). TA; Tibialis anterior, EDL; Extensor digitorum longus.

(b) Rate of change in absolute skeletal muscle weights (FL/GC). Sol; Soleus, Pla; Plantaris, Gas; Gastrocnemius. P-values from Student's t-tests are indicated as follows: *P < 0.05, **P < 0.01.

Supplementary Figure 2

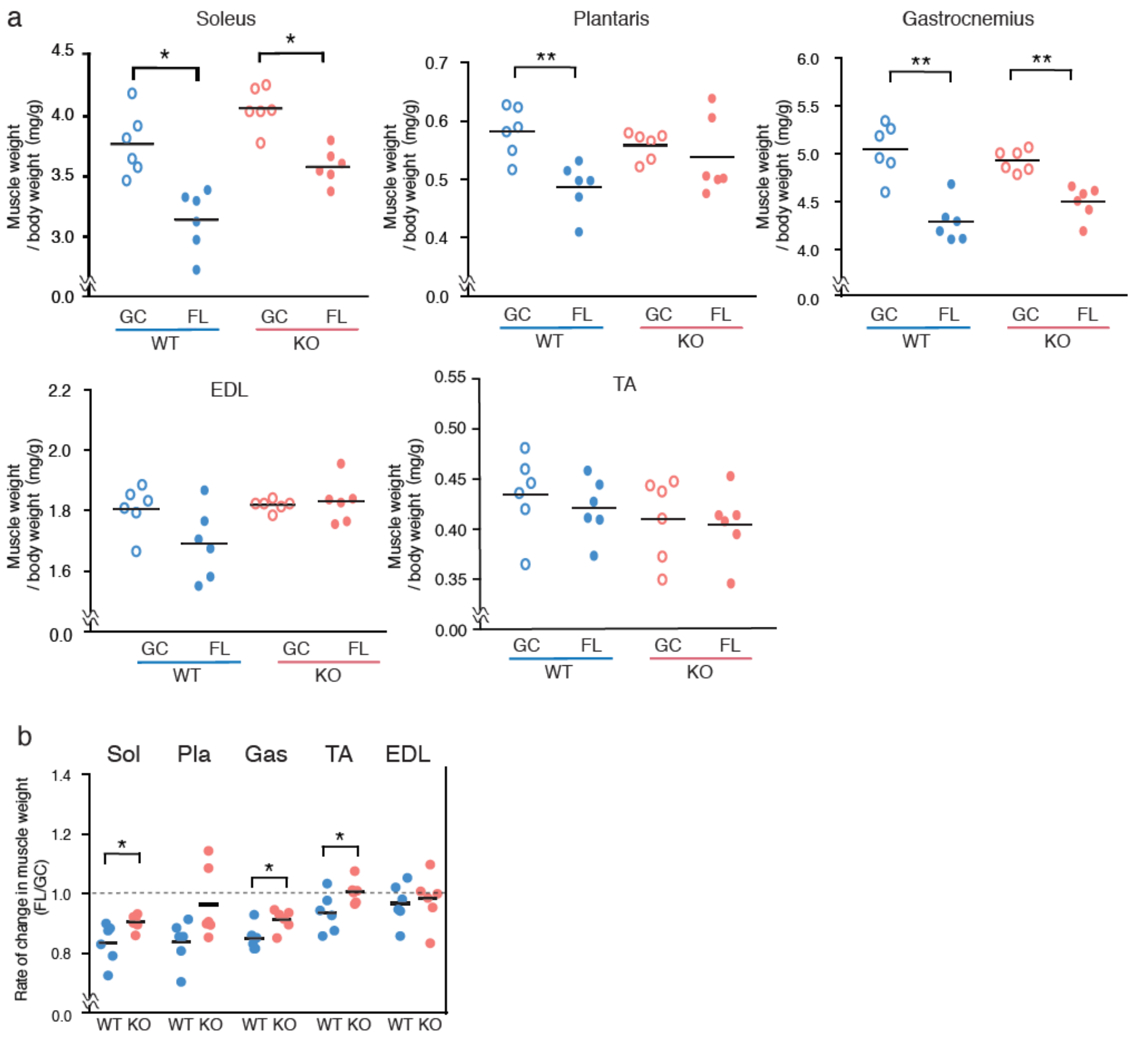

Supplementary Figure 2. Skeletal muscle weight normalised by body weight in space flight experiment

(a) Skeletal muscle weights normalised by body weight.

(b) Rate of change in normalised skeletal muscle weights (FL/GC). P-values from Student's *t*-test are indicated as follows: *P < 0.05, **P < 0.01.

Supplementary Figure 3

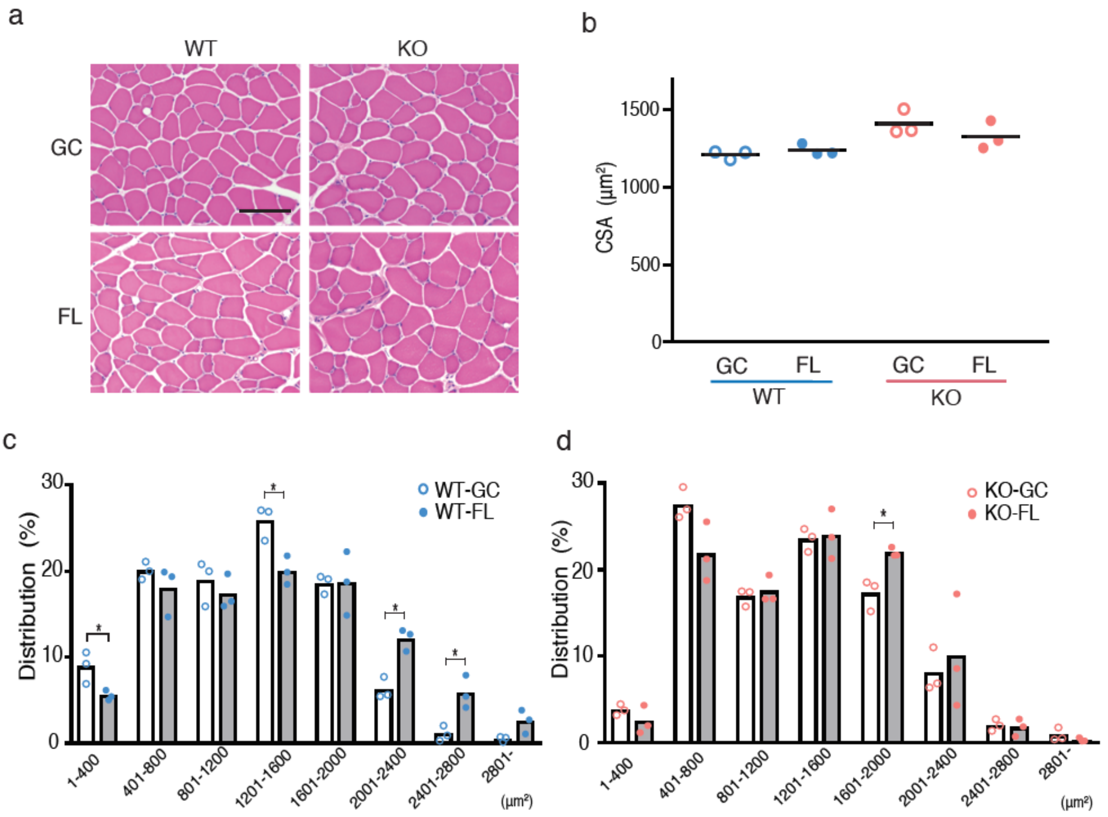

Supplementary Figure 3. Impact of KO on the EDL muscle of mice in a space experiment

(a) Haematoxylin-eosin staining of EDL muscle fibre sections. Scale bar = 100 µm.

(b) CSA of fibres EDL muscles in WT-GC (n = 3), WT-FL (n = 3), KO-GC (n = 3), and KO-FL (n = 3).

(c) Fibre area frequency distribution in WT-GC vs. WT-FL.

(d) Fibre area frequency distribution in KO-GC vs. KO-FL.

P-values calculated using Student's t-test are indicated as follows: *P < 0.05, GC vs. FL.

Supplementary Figure 4

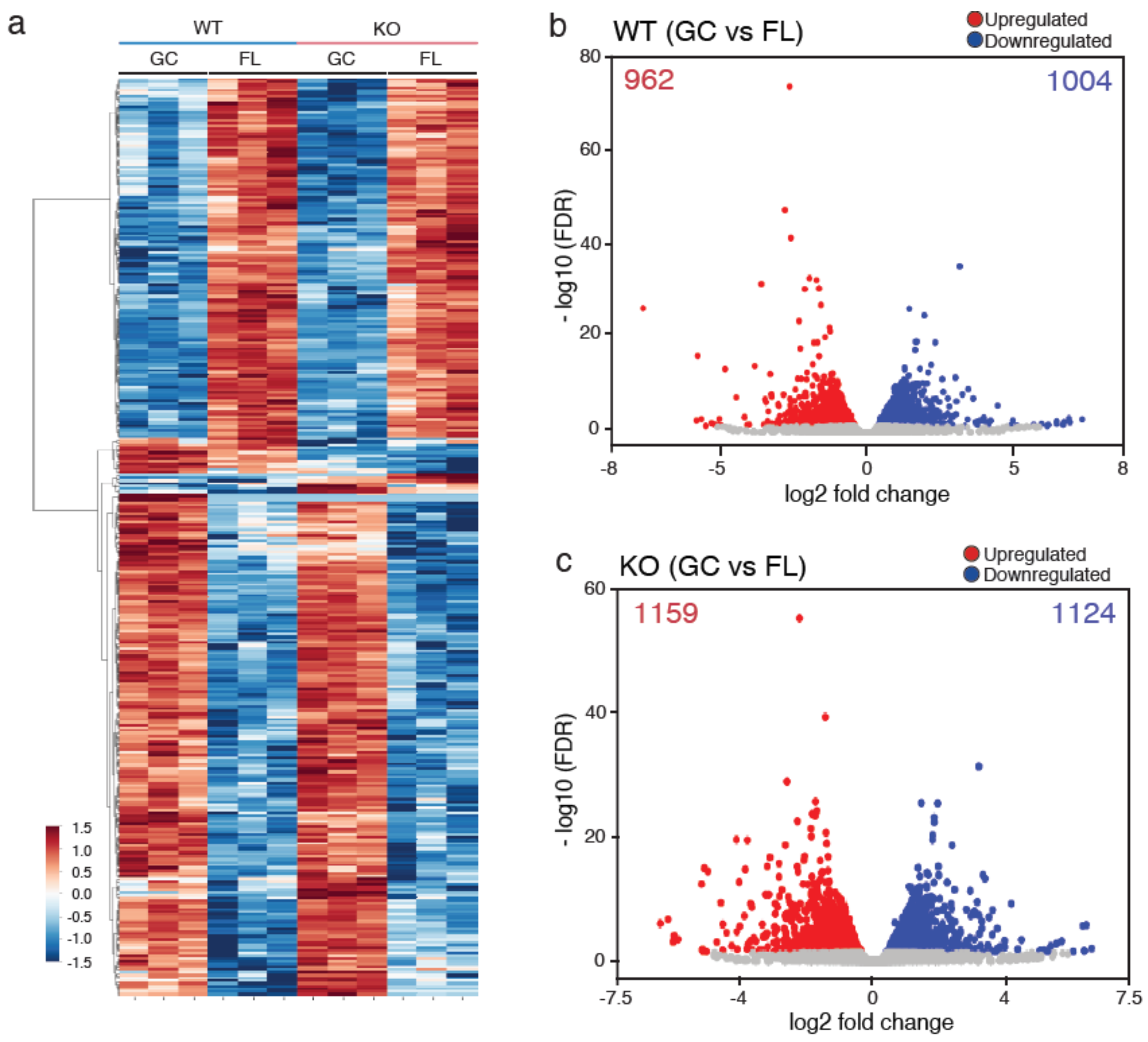

Supplementary Figure 4. Comprehensive analysis of gene expression by RNA-sequence

(a) Heatmap and hierarchical clustering of the gene expression values in the soleus muscles of WT-GC, WT-FL, KO-GC, and KO-FL mice including 1130 DEGs, ANOVA FDR-value < 0.05.

(b, c) Volcano plot of DEGs for each group; 962 upregulated genes and 1004 downregulated genes in WT (GC vs FL) (b); 1159 upregulated genes and 1124 downregulated genes in KO (GC vs FL) (c). edgeR test FDR < 0.05.

Supplementary Figure 5

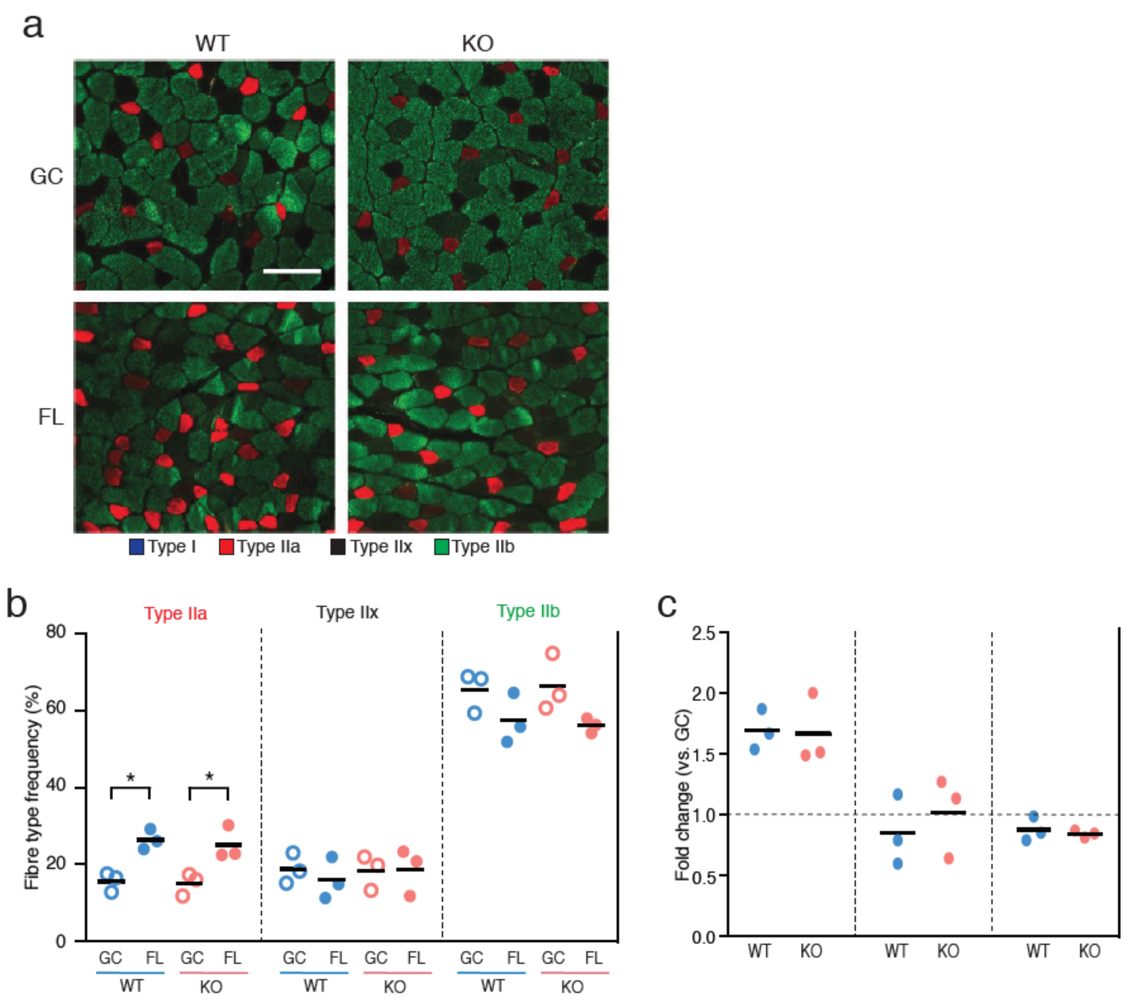

Supplementary Figure 5. Effect of KO on EDL fibre type transition

(a) Immunohistochemical staining for myosin heavy chain using BA-D5 (type I; blue), SC-71 (type IIa; red), and BF-F3 (type IIb; green) antibodies. Unstained fibres predicted to be type IIx (black). Scale bar = 100 µm.

(b) Frequency of each fibre type in WT-GC (*n* = 3), WT-FL (*n* = 3), KO-GC (*n* = 3), and KO-FL (*n* = 3) mice. P-values from Student's *t*-tests are indicated as follows. *P < 0.05, GC vs. FL.

(c) Comparison of FL fibre type frequency normalised by GC fibre type frequency.

Supplementary Figure 6

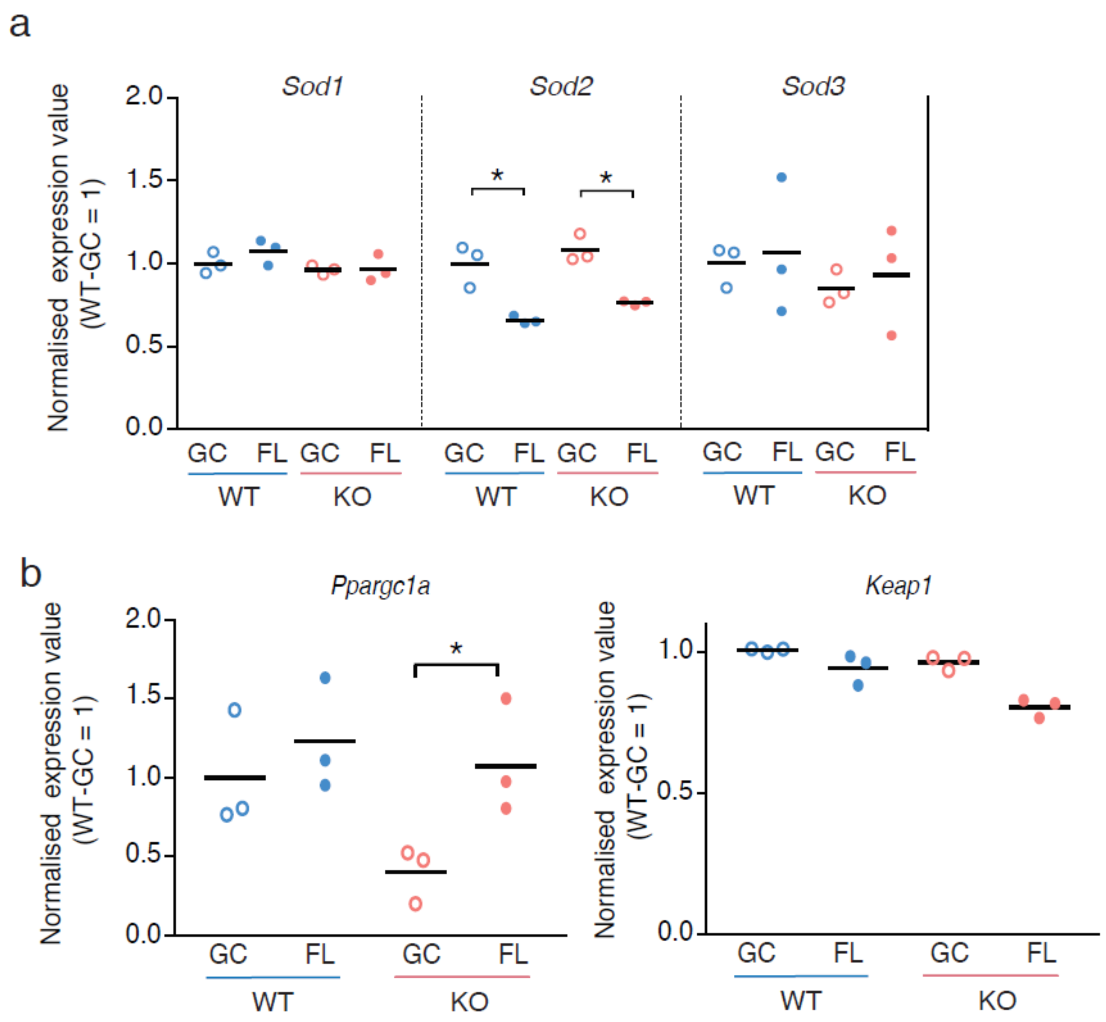

Supplementary Figure 6. Expression of NRF2-related genes

(a) Expression of *Sod* genes in soleus muscle.

(b) Expression of *Ppargc1a* and *Keap1* genes in soleus muscle. edgeR test *P < 0.05 (FDR-corrected).

Supplementary Table 1. Two-way ANOVA for soleus muscles

The panel shows the results of the two-way ANOVA for wet raw weight and CSA of soleus muscles.

Two-way ANOVA		
Wet raw weight		
Source of Variation	P value	Significant?
Interaction	0.8279	No
Gravity Factor	<0.0001	Yes
Genotypic Factor	0.1913	No
CSA		
Source of Variation	P value	Significant?
Interaction	0.9618	No
Gravity Factor	<0.0001	Yes
Genotypic Factor	0.0345	Yes

Supplementary Table 2. Fibre counts of each soleus and EDL muscles

Number of counted fibres for analysis of soleus and EDL muscles.

Soleus	Group	WT-GC (n = 4)					
	Mouse ID	#1	#2	#3	#4		
	Fibre counts	932	543	713	1415		
	Group	KO-GC (n = 5)					
	Mouse ID	#1	#2	#3	#4	#5	
	Fibre counts	521	802	829	667	720	
	Group	WT-FL (n = 6)					
	Mouse ID	#1	#2	#3	#4	#5	#6
	Fibre counts	776	1295	1113	1299	1055	1585
	Group	KO-FL (n = 6)					
	Mouse ID	#1	#2	#3	#4	#5	#6
	Fibre counts	688	1317	1407	829	1016	828

EDL	Group	WT-GC (n =3)		
	Mouse ID	#1	#2	#3
	Fibre counts	551	693	636
	Group	KO-GC (n =3)		
	Mouse ID	#1	#2	#3
	Fibre counts	443	478	553
	Group	WT-FL (n =3)		
	Mouse ID	#1	#2	#3
	Fibre counts	583	508	626
	Group	KO-FL (n =3)		
	Mouse ID	#1	#2	#3
	Fibre counts	512	956	485
