# Supplementary material for: Nuclear factor E2-related factor 2 (NRF2) deficiency accelerates fast fibre type transition in soleus muscle during space flight
Source: Commun Biol. 2021 Jun 24;4:787. doi: 10.1038/s42003-021-02334-4 (PMC8225765; doi:10.1038/s42003-021-02334-4)
Supplement: Supplementary file 2 — Description of Additional Supplementary Files [file 42003_2021_2334_MOESM2_ESM.pdf]

Description of Additional Supplementary Files

Supplementary Data 1~3 : All source data for graphs and charts presented in the main figures.

File Name: Supplementary Data 1
Description: Muscle weight

File Name: Supplementary Data 2
Description: CSA (soleus and EDL)

File Name: Supplementary Data 3
Description: Fibre type (soleus and EDL)
