# Supplementary material for: Nuclear factor E2-related factor 2 (NRF2) deficiency accelerates fast fibre type transition in soleus muscle during space flight
Source: Commun Biol. 2021 Jun 24;4:787. doi: 10.1038/s42003-021-02334-4 (PMC8225765; doi:10.1038/s42003-021-02334-4)
Supplement: Supplementary file 6 — Reporting Summary [file 42003_2021_2334_MOESM6_ESM.pdf]

nature research

Corresponding author(s): Takashi Kudo

Last updated by author(s): May 2, 2021

Reporting Summary

Nature Research wishes to improve the reproducibility of the work that we publish. This form provides structure for consistency and transparency in reporting. For further information on Nature Research policies, see our Editorial Policies and the Editorial Policy Checklist.

Statistics

For all statistical analyses, confirm that the following items are present in the figure legend, table legend, main text, or Methods section.

n/a	Confirmed	
☐	☒	The exact sample size (*n*) for each experimental group/condition, given as a discrete number and unit of measurement
☐	☒	A statement on whether measurements were taken from distinct samples or whether the same sample was measured repeatedly
☐	☒	The statistical test(s) used AND whether they are one- or two-sided<br>*Only common tests should be described solely by name; describe more complex techniques in the Methods section.*
☒	☐	A description of all covariates tested
☐	☒	A description of any assumptions or corrections, such as tests of normality and adjustment for multiple comparisons
☐	☒	A full description of the statistical parameters including central tendency (e.g. means) or other basic estimates (e.g. regression coefficient) AND variation (e.g. standard deviation) or associated estimates of uncertainty (e.g. confidence intervals)
☐	☒	For null hypothesis testing, the test statistic (e.g. *F*, *t*, *r*) with confidence intervals, effect sizes, degrees of freedom and *P* value noted<br>*Give P values as exact values whenever suitable.*
☒	☐	For Bayesian analysis, information on the choice of priors and Markov chain Monte Carlo settings
☒	☐	For hierarchical and complex designs, identification of the appropriate level for tests and full reporting of outcomes
☒	☐	Estimates of effect sizes (e.g. Cohen's *d*, Pearson's *r*), indicating how they were calculated

Our web collection on statistics for biologists contains articles on many of the points above.

Software and code

Policy information about availability of computer code

Data collection	Paired-end (2 × 36 bases) sequencing was performed using NextSeq500 (Illumina).
Data analysis	Sequence reads were mapped to the mouse reference genome (mm10). CLC Main Workbench (Version 7.7.3; Qiagen) for measurement of gene expression. iDEP.91 (http://bioinformatics.sdstate.edu/idep/) for PCA analysis, Python(Version 3. 8. 3) for generating heatmap,R studio (Version1.3.1056) for volcano plots, BZ-X800 (Keyence) for measurement of cross-sectional area.

For manuscripts utilizing custom algorithms or software that are central to the research but not yet described in published literature, software must be made available to editors and reviewers. We strongly encourage code deposition in a community repository (e.g. GitHub). See the Nature Research guidelines for submitting code & software for further information.

Data

Policy information about availability of data

All manuscripts must include a data availability statement. This statement should provide the following information, where applicable:

- Accession codes, unique identifiers, or web links for publicly available datasets
- A list of figures that have associated raw data
- A description of any restrictions on data availability

All data needed to evaluate the conclusions in the paper are present in the paper or the supplementary materials.

Field-specific reporting

Please select the one below that is the best fit for your research. If you are not sure, read the appropriate sections before making your selection.

[x] Life sciences [ ] Behavioural & social sciences [ ] Ecological, evolutionary & environmental sciences

For a reference copy of the document with all sections, see nature.com/documents/nr-reporting-summary-flat.pdf

Life sciences study design

All studies must disclose on these points even when the disclosure is negative.

Sample size	A mouse space experiment: 3-6 in each group.
Data exclusions	Due to damage to the frozen tissue samples, ID Nos. 3 and 6 of WT-GC and ID No. 6 of KO-GC could not be analyzed.
Replication	All experiments included contain independent biological replicates.
Randomization	Data were not analyzed with randomization.
Blinding	Investigators were not blinded during experiment.

Reporting for specific materials, systems and methods

We require information from authors about some types of materials, experimental systems and methods used in many studies. Here, indicate whether each material, system or method listed is relevant to your study. If you are not sure if a list item applies to your research, read the appropriate section before selecting a response.

Materials & experimental systems

n/a	Involved in the study	
x		Antibodies
x		Eukaryotic cell lines
x		Palaeontology and archaeology
	x	Animals and other organisms
x		Human research participants
x		Clinical data
x		Dual use research of concern

Methods

n/a	Involved in the study	
x		ChIP-seq
x		Flow cytometry
x		MRI-based neuroimaging

Animals and other organisms

Policy information about studies involving animals; ARRIVE guidelines recommended for reporting animal research

Laboratory animals	Nrf2-KO (Nfe212tm1Ymk) and age-matched WT male mice in the C57BL/6J background were used.
Wild animals	No wild animals was used in this study.
Field-collected samples	This study does not involve samples collected from the field.
Ethics oversight	All animal experiments were approved by the Institutional Animal Care and Use Committees of the University of Tsukuba (No. 18–451), JAXA (Protocol Numbers 017–001 and 017–014), NASA (Protocol Number FLT- 17-112), and Explora BioLabs (Study Number: EB15-010C), and conducted according to the related guidelines and applicable laws of Japan and the United States of America.

Note that full information on the approval of the study protocol must also be provided in the manuscript.
